# The silent burden: a landscape analysis of common perinatal mental disorders in low- and middle-income countries

**DOI:** 10.1186/s12884-022-04589-z

**Published:** 2022-04-20

**Authors:** Shanon E McNab, Sean L Dryer, Laura Fitzgerald, Patricia Gomez, Anam M. Bhatti, Edward Kenyi, Aleefia Somji, Neena Khadka, Suzanne Stalls

**Affiliations:** MOMENTUM Country and Global Leadership, Washington, DC, USA

**Keywords:** Maternal mental health, Perinatal mental health, Maternal health, Common perinatal mental disorders, Social determinants, Child health

## Abstract

**Background:**

Mental health has long fallen behind physical health in attention, funding, and action—especially in low- and middle-income countries (LMICs). It has been conspicuously absent from global reproductive, maternal, newborn, child, and adolescent health (MNCAH) programming, despite increasing awareness of the intergenerational impact of common perinatal mental disorders (CPMDs). However, the universal health coverage (UHC) movement and COVID-19 have brought mental health to the forefront, and the MNCAH community is looking to understand how to provide women effective, sustainable care at scale. To address this, MOMENTUM Country and Global Leadership (MCGL) commissioned a landscape analysis in December 2020 to assess the state of CPMDs and identify what is being done to address the burden in LMICs.

**Methods:**

The landscape analysis (LA) used a multitiered approach. First, reviewers chose a scoping review methodology to search literature in PubMed, Google Scholar, PsychInfo, and Scopus. Titles and abstracts were reviewed before a multidisciplinary team conducted data extraction and analysis on relevant articles. Second, 44 key informant interviews and two focus group discussions were conducted with mental health, MNCAH, humanitarian, nutrition, gender-based violence (GBV), advocacy, and implementation research experts. Finally, reviewers completed a document analysis of relevant mental health policies from 19 countries.

**Results:**

The LA identified risk factors for CPMDs, maternal mental health interventions and implementation strategies, and remaining knowledge gaps. Risk factors included social determinants, such as economic or gender inequality, and individual experiences, such as stillbirth. Core components identified in successful perinatal mental health (PMH) interventions at community level included stepped care, detailed context assessments, task-sharing models, and talk therapy; at health facility level, they included pre-service training on mental health, trained and supervised providers, referral and assessment processes, mental health support for providers, provision of respectful care, and linkages with GBV services. Yet, significant gaps remain in understanding how to address CPMDs.

**Conclusion:**

These findings illuminate an urgent need to provide CPMD prevention and care to women in LMICs. The time is long overdue to take perinatal mental health seriously. Efforts should strive to generate better evidence while implementing successful approaches to help millions of women “suffering in silence.”

**Supplementary Information:**

The online version contains supplementary material available at 10.1186/s12884-022-04589-z.

## Background

Mental health has long fallen behind physical health in attention, funding, and action, despite nearly one billion people throughout the world living with some form of mental illness [1, 2]. However, the universal health coverage (UHC) movement and COVID-19 have brought mental health to the forefront, and the maternal, newborn, child, and adolescent health (MNCAH) community is looking to understand how to provide women effective, sustainable care at scale. People with mental disorders experience disproportionately higher rates of disability and mortality, and more than 80% of people living with mental health conditions reside in low- and middle-income countries (LMICs) [3, 4]. The treatment gap in LMICs is estimated at nearly 90% and is compounded by the poor quality of most available treatment [4, 5]. However, evidence generation, as well as the framing of mental health and definitions of mental health conditions, are grounded in high-income countries rather than in LMIC settings where the need is the greatest [6]. The absence of mental health from global MNCAH programs is particularly concerning, given the increasing awareness of the intergenerational impact of common perinatal mental disorders (CPMDs) [7]. The need for context-appropriate, high-quality maternal mental health programming is further amplified for vulnerable groups—adolescents, women experiencing gender-based violence (GBV), women living in humanitarian settings, women experiencing obstetric trauma, or those living in poverty. Global level action plans and implementation guidance have not yet been translated into evidence-based maternal mental health programs that effectively reach women at scale.

This paper presents findings of a landscape analysis (LA) conducted by a team from USAID’s MOMENTUM Country and Global Leadership (MCGL) to better understand the current context of maternal mental health programs in LMICs [8]. It discusses the intergenerational impact of CPMDs, risk factors, gaps in knowledge, and promising implementation strategies that, if sustainably scaled in real world settings, could help ensure women receive the quality physical and mental health care that is their human right.

## Methods

A multitiered approach was used for our landscape analysis that included a modified scoping review, qualitative interviews, and a policy review to address the aims and build a broad understanding of the current literature on perinatal mental health (PMH) in LMICs and to examine the relationship between PMH and MNCAH outcomes, including promising interventions and implementation strategies.

The Institutional Review Board of the Johns Hopkins Bloomberg School of Public Health determined that this activity did not qualify as human subjects research as defined by the DHHS regulations 45CFR 46.102. Verbal consent was approved by the IRB as the project was deemed minimal risk. Because of COVID-19 precautions, as well as practical considerations, there were no in-person interactions between staff and participants; instead, all interviews were conducted via Zoom. Participants provided verbal consent, and no participants declined to consent.

### Scoping review

The modified scoping methodology iteratively refined the literature search at each of its five stages, per Arksey and O’Malley [9]. This approach was chosen because it supports the identification of knowledge gaps by setting research agendas and informs decision-making while allowing for an iterative approach [10].

### Stage 1: identifying the research question

The following research questions were identified:

- What effects do common perinatal mental disorders have on maternal wellbeing and newborn/child health and development outcomes in LMICs?
  - º What are the risk and protective factors for common perinatal mental disorders?
- What interventions have been implemented in LMICs to prevent, identify, manage, and treat perinatal mental health conditions and/or improve child outcomes?
  - º What were the successful mechanisms, in what context, that could be tested and scaled in other settings?
  - º What gaps in knowledge and implementation remain?

### Stage 2: identifying relevant resources

Relevant resources were identified through searching electronic databases of the published literature (PubMed, Google Scholar, PsychInfo, and Scopus) with the following search terms: maternal mental health, child/newborn outcomes, prenatal depression, postnatal depression, antenatal depression, postpartum depression, perinatal depression, maternal depression, perinatal mental health, stillbirth, intervention studies, low-and-middle income, and developing country. The team used the same search terms in grey literature (USAID Development Experience Clearinghouse, websites of organizations working in PMH) for relevant program, research, and testimonial documents. Iterative keyword searches, forward citation tracking, and the inclusion of resources from key informant libraries were also done to identify additional resources to help better frame the context of PMH. Targeted searches were iteratively added during the review to better understand emerging issues of relevance: humanitarian and fragile settings, family planning, adolescents, stillbirth/perinatal loss, gender-based violence, and COVID-19. The following criteria were used for exclusion: Resources published before 2005, published in language other than English, focused on outcomes of children older than 3 years, about substance abuse only, abstracts, protocols, high income country focus, and “global” studies without LMIC representation^1^. The searches were conducted December 2020 through June 2021.

### Stage 3: resource selection

**Fig. 1:**
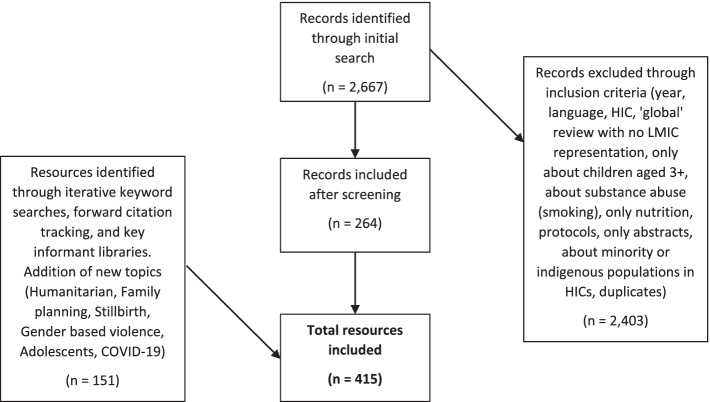
Resources flow chart

Resources (articles, reports, studies, policies, etc.) were iteratively screened, reviewed, and selected by at least two authors through abstract review and then full document review. Please see Fig. 1 for the flow chart.

### Stage 4: charting the data

A tool was created to extract information from 12 categories of literature: perinatal mental health, measurement, family planning, humanitarian, newborn/child, gender and GBV, stillbirth, nutrition, COVID, grey literature, interventions, and adolescents. The data extraction themes included categories for context, risk factors, interventions, gaps in evidence, and recommendations.

### Stage 5: collating, summarizing, and reporting results

Included resources were analyzed across themes and sub-themes mentioned above. Excerpts from each paper were coded based on their relevance to one or more themes. Two reviewers (SM and SD) independently reviewed four sample resources and compared all extractions. Discrepancies were discussed and definitions for themes were refined. The remaining team members reviewed one or two sample resources with these reviewers, and disagreements led to further refined definitions.

### Qualitative data

Key informant interviews (KIIs) were conducted with 44 experts identified through the literature and snowball sampling and who were working in the fields of mental health, maternal, newborn and child health, adolescent health, humanitarian and fragile settings, nutrition, gender-based violence, stillbirth and perinatal loss, advocacy, and implementation research. In addition, two focus group discussions (FGDs) were conducted with experts identified through the Inter-Agency Working Group on Reproductive Health in Crises to understand the status of PMH in refugee camps, conflict settings, and fragile states. Interview and FGD guides were drafted specifically for our study, and they are provided as Additional files 1 and 2. All KIIs and FGDs were conducted in English via Zoom by a team member between December 2020 and June 2021 and were 60–90 min in length. Verbal consent was obtained before audio recording began. Audio files were transcribed verbatim, reviewed for clarity and accuracy, and uploaded into Dedoose.

An inductive process used the “codebook” approach to thematic analysis [11]. Structural coding was used to identify top-level themes related to the research questions, and data within each theme re-coded to generate sub-themes iteratively, shifting from “domain summary” themes toward “pattern” themes, as defined by Braun et al., with each cycle [11, 12]. This cycle was repeated as many times as required to allow effective areas of consensus and controversy to be identified and reported. The first 25 interview transcripts were reviewed in full by one author (SD) and used to identify top-level and sub-themes, which were defined and shared with another author (SM), who read 12 transcripts for consensus. Because of the wide breadth of the research objectives, 46 sub-themes were generated in the first tier of sub-themes and further iteration was not required. All transcripts were reviewed and coded in Dedoose based on these themes. Throughout the coding process, more detailed findings or relationships between codes were captured as they were identified using analytic memos.

### Policy review

The team conducted a documentary analysis with a purposive sample of 19 LMIC countries^2^ to assess specific national policies and policy gaps [13]. The team searched WHO Mind Bank, Ministry of Health websites, WHO Mental Health Atlas, and citation-mined literature. The team also contacted project country staff. A data extraction tool was used to capture relevant PMH policy details (e.g., existence of national level mental health policy). The content analysis was conducted by EK.

## Results

### Prevalence and impacts on women and children

PMH includes mental health during the perinatal period. The period generally commences with pregnancy, but may be defined as extending up to 2 years after delivery [14, 15]. Within PMH are a set of CPMDs—depression, anxiety, and somatic disorders [16]. PMH is inextricably linked with a woman’s physical health during the perinatal period and has important implications for her long-term mental and physical health, functioning, and quality of life [17]. Women with CPMDs face numerous health consequences because of the challenges related to nutrition, substance abuse, adherence to treatment regimens for physical health conditions such as HIV, and access to medical care, and women with a history of major postpartum depression have a 25% risk of recurrence in a subsequent pregnancy [18–21]. Maternal anxiety is a known risk factor for both suicide and depression [22, 23]. A global systematic review estimated that 20% of mortality in the year after childbirth occurs by suicide [24]. Antenatal mood disorders are associated with increased risk of preterm birth and pre-eclampsia, which are associated with increased all-cause mortality and death from cardiovascular disease later in life [19, 25].

PMH conditions do not only affect women, they affect the physical, emotional, and neurological development in newborns and children [26]. Depressed mothers from LMICs have higher risks of preterm births and low-birthweight babies [27]. Adverse birth outcomes set the stage for higher mortality for children. Studies have shown increased risk of death for children whose mothers have postnatal depression [28, 29]. The effects on children extend beyond birth outcomes and lowered survival rates. Approximately 7.2 million cases of stunting in LMICs have been estimated to be attributable to psychosocial factors, which include CPMDs [30]. Systematic reviews indicate that CPMDs are negatively associated with children’s motor, cognitive, language, behavior, and global development [31, 32]. They increase incidence of diarrhea and acute respiratory illness, generate insufficient milk supply (and subsequent ceasing of breastfeeding), and result in poorer bonding [33–35]. Key informant interviews confirmed these findings and emphasized the link between CPMDs and poor child outcomes:

*“A key intervention for children with diarrhea is to teach mothers to give oral rehydration solution. Some mothers learn very quickly, while others are not as engaged. When I saw depressed women in the hospital, I immediately made a link between the two things. That became my first research—to look at the associations of maternal depression and infant growth and development disease. And lo and behold, we found very strong associations between maternal mental health and birth weight, diarrheal disease, breastfeeding duration. So maternal depression is not only bad for the mum, it’s bad for the child*.” — Researcher, LMIC

**Fig. 2:**
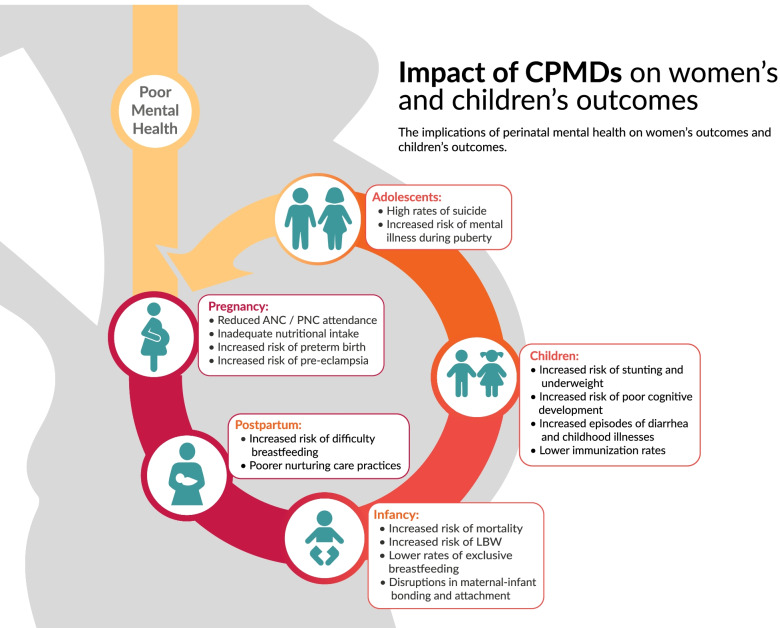
Impact of CPMDs

Please see Fig. 2 for a graphic depiction of how CPMDs affect both mother and baby.

### Risk factors

The risk factors of maternal mental illness for women living in LMICs stem from broader social factors, such as economic or gender inequality, to more individual experiences, such as a history of stillbirth and genetic predisposition. The likelihood and severity of the PMH condition is likely to vary depending on the cultural context and social factors that are often beyond a woman’s control [22, 36, 37]. Cultural, sociopolitical, economic, and environmental factors influence all aspects of a woman’s life, including her access to services, social support, freedom from gender-based violence, nutritional status, and her baby’s ability to grow and thrive [22, 27, 36, 38–40]. The LA found inequitable gender norms that limit autonomy, poverty, and intimate partner violence (IPV) to be the most significant drivers of PMH conditions and prevent women from seeking and accessing care [37–39, 41]. As one key informant explained:

*“A lot of women come [to] Joburg for a better life or a better opportunity. You know, and then it doesn’t happen, then you end up living in an informal settlement, or a shack, you can’t get a job. So, these women end up in these relationships with men, where the financial power that these men have over these women, [that] they have to be in these relationships. So, number one is poverty. Number two is intimate partner violence, gender-based violence*.” *—* Clinician, LMIC

Many of these risks have been further increased by the COVID-19 pandemic, with women more likely than men to lose their jobs, an increase in child and forced marriages, and increased emotional, physical, and sexual violence [42–44]. A study in China demonstrated an increase in pregnant women’s depressive symptoms from 26% pre-pandemic to 34.2% during the pandemic [45].

### Interventions

#### What interventions are being implemented?

PMH service guidelines, manuals, initiatives, and projects are being introduced and implemented throughout LMICs. Some initiatives work in communities and others in health facilities. Most approaches involve task shifting from specialized mental health professionals to more generalized health workers, involve a stepped-care model in which more severe cases are referred, and meet women where they seek care/support [46–49]. Most interventions use a local adaptation of cognitive behavioral therapy, but others include problem solving therapy, behavioral activation, group-based programs, family-based programs, parenting skills, mother-baby sessions, play-based support, etc. [18, 50–54]. Many interventions adapt existing interventions to better meet the specific needs of women in the perinatal period and/or are integrated into existing services targeting women during this period, such as antenatal care (ANC) or postnatal care (PNC) [46, 55, 56]^3^. Please see Table 1 for details about specific programs and interventions, what they include, and related evidence of improved outcomes.

**Table 1:** Programs, interventions, and outcomes

Program/Intervention	Context	Evidence on PMH outcomes	Evidence on child outcomes
**Group psychoeducation**^**a**^	Delivered in India by local women [57].	Improved depression symptoms.	Improved exclusive breastfeeding rates Reduced rates of infectious illnesses.
	Delivered in China by researchers [58].		
	Delivered in Iran by unspecified providers [59].		
**Thinking Healthy Program (adapted cognitive behavioral therapy)**	Delivered in rural Pakistan by Community Health Workers (CHWs) [46].	Improved depression symptoms and care seeking.	Improved exclusive breastfeeding rates and reduced rates of child infectious illnesses.
	Delivered in India by peers [60].	Improved depression symptoms.	
	Delivered in slums in Pakistan when delivered in groups by psychologists and combined with child development education [61].		
**Group cognitive behavioral therapy**	Delivered in South Africa by mentor mothers [53].	Improved depression symptoms.	
	Delivered in Iran by specialists [62].	Improved anxiety symptoms.	
**Interpersonal psychotherapy**	Delivered in China by midwife educators [63, 64].	Improved depression symptoms.	
	In Uganda, within peer groups with trained facilitators [65].		
**Newborn care educational program**	Delivered in South Africa by local women [66].	Improved depression symptoms.	Improved child weight-for-age.
	Delivered in Jamaica by CHWs [67].		
	Delivered in Nepal by unspecified providers [68].	Improved anxiety symptoms.	

^a^Interventions listed under “Group psychoeducation” and “Newborn care educational program” may differ in exact content

### Implementation components

Using a modified version of the consolidated framework for implementation research, the LA identified core components of successful interventions in LMICs at the community and health facility levels [69].

### Implementation core components at community level

#### Well supported task-sharing

To leverage existing cadres, and address the treatment gap, the global health community has endorsed task sharing to non-specialists [48]. Ensuring continuous oversight and support is needed to know whether interventions are implemented as intended [70]. Two main delivery mechanisms have shown success in expanding good quality PMH services in LMICs: use of existing community health workers (CHWs) and peers who are already trusted agents in the community. Well documented interventions, including the Thinking Healthy Program, have found that a cascade model to train and supervise existing CHWs in some form of talk therapy improved outcomes for women and babies. Women had less disability, improved functioning, and were more likely to use contraception [71]. Infants of treated women had fewer episodes of diarrhea and were more likely to be immunized [71]. The Thinking Healthy Program also found that female peers, rather than CHWs, who facilitate sessions about PMH may be preferable for women with less chronic or severe depression and a good first step in the stepped care approach [72]. The low cost eases introduction, implementation, and scale-up. Women value other women who know their culture, speak their language, and understand community stigma [73].

#### Stepped care (inclusive of a clear referral system)

Stepped care—providing gradually progressive mental health services and expertise based on individual need—is an effective approach to ensuring as many women as possible receive psychosocial support and have access to basic PMH services, while those with need can access more specialized treatment [74]. CHWs and other frontline workers cannot solve all CPMDs: The initial “step” is the least resource intensive intervention and prevents an overflow of referrals to health facilities if appropriately implemented in communities [48, 75]. Stepped care requires the existence of services for more complicated cases with clear referral protocols and pathways [75]. Some key informants who work in mental health questioned the reality of referral for more advanced care: “*... Even when you implement* mhGAP*, you’re still in the same situation that the person is largely being referred to services that don’t exist”* (Donor staff KII).

#### Talk therapy: cognitive behavioral therapy, behavioral activation, problem-solving therapy

Talk therapy, or psychotherapy, describes a range of dialogue-based treatment options for mental illness in which a trained provider examines clients’ thoughts and behavior to identify changes that alleviate symptoms [76]. In LMICs, talk therapy has focused on approaches, such as cognitive behavioral therapy, with evidence that they can be delivered by non-specialist health workers [77]. Training must be appropriate for the female session facilitators (CHWs, community grandmothers, or peers) (KII 39). Facilitators must be comfortable with the session material, believe it will resonate within their community, and have a supervision strategy to assure quality over time.

#### Contextualized language for CPMDs

PMH programs must be mindful of the cultural relevance of mental illness and the distinct idioms used. How mental health conditions are expressed are highly variable and tools that rely on specific language constructs can fail to adequately reflect what is happening emotionally for a woman. Recognizing this challenge, Honikman et al. suggested these strategies to ensure cultural coherence in language and tools: a) formative research; b) qualitative enquiry with stakeholders; c) pilot testing of trainings and psychosocial interventions; d) adapting detection methods and screening tools to include emic concepts; and e) using community-based providers to deliver elements of the interventions [40].

#### Strengthen GBV services

The relationship between GBV and mental health is well established; GBV threatens the health and survival of women and babies during the perinatal period [78, 79].

“*Perinatal mental health troubles … [are] associated with IPV. A lot of it is understandably about disempowerment, lack of control, and that is just reemphasized over and over again in other problems women face. So, if you have no control over your fertility, then you will feel disempowered and out of control. If you have an abusive partner, you will feel controlled by him. If you are very poor and can’t decide what to spend your money on, whether your kids go to school, you’ll feel disempowered, all of these would be factors for depression, and other adverse mental health [conditions].”* — Researcher with expertise in MMH, adolescent, and gender norms

#### Anti-stigma initiative/advocacy/campaign

Because stigma can prevent women and families from seeking care and providers from providing care, PMH programs should work with communities to co-create anti-stigma activities. Approaches to reduce mental illness related stigma and discrimination are ripe for further evidence [80]. There are tools, such as the Discrimination and Stigma Scale (DISC) for initial assessment, networks of researchers, and a newly created *Lancet Commission on stigma and discrimination in mental health* that is expected to publish a report in 2022. This report will include a global literature review on the effectiveness and cost-effectiveness of interventions to reduce stigma and discrimination from which to draw guidance [81, 82]. One key informant emphasized the importance of addressing stigma – that if this were not an essential part of the larger response to PMH, the impacts could be devastating:

*“I think that we have to think very carefully about solutions that don’t make the problem worse. So mental health problems, psychiatric labeling, are highly stigmatizing in most low- and middle-income countries, they constitute enormous barriers to the likelihood that a woman would ever accept such a label, be willing to have assistance for it. So there’s an important, I think, consideration around stigma and how that influences what might be the most helpful response.”* — Global expert on MMH

### Implementation core components at health system level

#### Pre-service training/modules on mental health

Institutionalized education during provider pre-service training rarely includes CPMD-specific modules. Several key informants noted the need to train incoming health care workers (HCWs) about mental health, de-stigmatize it, and give HCWs the skills to help women facing mental health issues (KII 13, KII 14).

#### Trained and supervised health care providers

Various HCW cadres have been trained and supervised to provide women with mental health services. Often, women’s first consistent interface with the health system in LMICs is ANC; thus, ANC is seen as a good place to make PMH services available. Depending on context and women’s preferences, junior public health nurses, midwives, or psychosocial workers have been trained and supervised to provide either one-on-one or group CPMD services for women attending ANC or PNC (KII 12, KII 22) [83]. Whichever cadre facilitates these interventions must have a clear referral pathway for mental health conditions beyond their scope of practice and have the skills to address imminent risk of self-harm or suicide [84].

#### Screening process

Several interventions use a screening tool at the facility level. The right tool, for the right purpose, must be chosen and adapted to the local community. Lasater et al. noted, “Common perinatal mental disorders may look different, or may be experienced and communicated differently, depending on local cultural contexts,” and warned against presuming that what works in one context will in another [17]. Screening tools should be integrated into the existing system, rather than using a new system, to avoid further burdens on health care providers [18]. Importantly, experts warn against screening without confirmation by clinical assessment before offering a diagnosis or initiating treatment. In addition, many experts promote universal approaches to improve mental health of all women—not only those who meet diagnostic criteria (KII 2, KII 11). A single use of a screening tool may not accurately reflect the mental health continuum of a perinatal woman.

#### Mental health support for health care providers

Several key informants expressed that the mental health of HCWs themselves is often ignored (KII 13, KII 14). The onset of COVID-19 has exposed the glaring needs and consequences of not supporting HCWs during a pandemic: burnout, absenteeism, leaving the profession entirely, depression, and increasingly death by suicide. The push for self-care has been important, but systems to help manage the mental health of HCWs in resource-deprived settings are crucial. Some interventions are attempting to address provider mental health through debriefing sessions and external counselors for groups and individuals (KII 23).

*“I think there are double standards around mental health in general across the board in our society. I don’t see leaders educated on mental health. I don’t see that proper messaging in their language. I don’t see educators, whether they are medical specialists or not. I don’t see that happening in schools, by teachers, and in general in … fields like fashion and … other kinds of industries we are not seeing proper messaging. So I would not … put the burden of it on providers. Providers are part of a larger system, and they are merely mirroring to us what is being mirrored to them and what they are internalizing.” —* LMIC Researcher

#### Clear and accessible referral process

Referral pathways that do not rely on personal cellular data or providers’ personal funds and that are clear and easy for women to follow are essential. Three physician KIIs noted that many women get “lost” in referrals and leave health facilities without getting the care they need (KII 8, KII 9, KII 20).

#### Respectful maternity care

Women’s experiences of disrespect and abuse during pregnancy and childbirth impact their mental health. Women who experienced a previous loss and/or obstetric trauma were more likely to experience a CPMD than those who did not [85–87]. Yet, respectful maternity care (RMC) was rarely mentioned as part of integrated PMH and maternity care.

#### Link with and strengthen GBV services at facilities

GBV services should be strengthened and based on local gender norms. GBV services have found links within and beyond (education, protection, legal) the health sector. The ability to link with services that have many parallels with CPMDs needs could further strengthen the support system for women [88, 89]. Key informants discussed the importance of addressing the comorbidities associated between GBV and CPMDs:

*I think when you talk about sort of major risk factors like support and intimate partner violence and things like that, that’s all things that we fold into a program… that addresses comorbidity. And so, when we think about it, it’s not separate. You know, when you think about a woman who’s getting beaten and [is] emotionally berated and she’s depressed, that’s sort of just all one big bucket that we can fix with a true multi-problem intervention. —* Researcher, clinician

### Adaptable periphery components

Within interventions, adaptations are required to work in any one setting. The LA identified three aspects of PMH program implementation that varied significantly according to the implementation context. The first is the selection of the trusted delivery agent. Many studies that looked at the feasibility and acceptability of a PMH intervention examined the qualities women wanted in their service providers and with whom women would feel most comfortable sharing information about their condition. Many studies found that women preferred that their provider or CHW was a woman from their community—understanding the language, culture, and social norms [49, 70]. But the responses are diverse, and the ultimate decision should be based on a close understanding of the community. The second is how to fairly compensate the cadre implementing a PMH intervention. This compensation varied. Some relied on the altruistic nature of CHWs or peers, and others provided compensation or linked with existing CHW compensation structure. Adding responsibilities and skills to an existing cadre raises questions in any community-based intervention and must be answered based on the context [90]. The third adaptable component was the tools used to screen and measure CPMD. The use and usefulness of measurement tools has varied widely. Screening tools at facilities can be seen as one more “job” for HCWs to take on and will not be used if facilities are overwhelmed. Studies and evaluations use tools to show change in women’s CPMDs and intervention impact. The need for the tool and the purpose of the data should be grounded in what is locally feasible and acceptable (KII 22).

### The state of perinatal mental health policies

All 19 countries looked at reported stand-alone mental health policies, but explicit perinatal mental health policies were lacking. However, even where a mental health policy exists, inadequate financial and human resources, lack of dissemination, lack of prioritization, and unclear roles and authority hamper implementation [91, 92]. PMH has not received financed and articulated prioritization at the national policy level.

## Discussion

The findings of the LA suggest that several critical considerations can strengthen the acceptability, efficacy, and sustainability of interventions to improve maternal mental health. First, implementation context and social determinants of health must be prioritized when planning and executing interventions for CPMDs. Additionally, interventions must be integrated into existing strong health (or other) platforms. While the evidence supports the importance of these considerations, gaps remain in research and implementation learning related to *how* to best integrate and implement successful interventions, presenting a clear call for future research and continued sharing of implementation lessons learned. Our LA found that, too often, programs or interventions invest insufficient time in assessing and incorporating contextual factors and that there remains room for more inclusive and women centered research to better include evidence from LMICS into the conversation about PMH.

Taking the time to fully understand the context of women’s lives, as well as the numerous social determinants that affect women’s mental wellbeing, is crucial in the design and implementation of successful PMH interventions. Before introducing any new intervention, thorough assessments should be conducted to identify community assets and barriers and develop a deep understanding of women’s needs, priorities, and preferences. Such assessments should include qualitative interviews and FGDs with community actors to learn about existing stigmas, explore how communities define CPMDs in their own language, and identify existing health programs/platforms that can be leveraged. Learnings from these assessments should then inform key program or intervention decisions.

Findings from the LA also underscored the importance of addressing social determinants of health in the communities in which programs or interventions will be implemented. In particular, programs should include gender analyses to contextualize local gender and power norms and understand how best to identify and support women who are currently experiencing or have experienced GBV. Our findings also suggest that linking PMH interventions with the growing RMC movement could present an opportunity to address risk factors for CPMDs and protect women’s mental wellbeing through honoring contextually specific language related to childbirth, valuing women’s expectations for care, and promoting respectful treatment for women and newborns around the time of birth. Lastly, if interventions are responsive to implementation contexts and the ways in which women express mental illness and suffering, resulting screening and assessment tools may be even more accurate and useful for women and providers.

In addition to ensuring interventions account for key contextual factors and social determinants of health, the LA found that perinatal mental health interventions should not be created in parallel, or in a silo, from the important work being done more broadly to improve MNCH outcomes. Rather, the imperative to integrate CPMD interventions into existing platforms came out clearly. Integration should be considered at two levels. The first level is the *integration of mental health as a priority across health sectors* (MNCAH, nutrition, HIV, etc.) and beyond health (education, WASH, protection, etc.), rather than siloed by vertical funding and programming structures. The LA findings called for integrated programming, funding, communities of practice, and research. UNICEF’s Caring for the Caregiver, Helping Adolescents Thrive, and the MAMI Care Pathway Package are examples of integrated care packages using the PMH tools discussed earlier [93–95]. Programs must also integrate with others focused on issues such as economic empowerment, food security, and gender transformation, given their outsized impact on PMH. The second level is the *integration of PMH into primary health care, from centralized institutions to primary health care and communities*. This need has been promoted by WHO, Lancet Commissions, and international mental health societies [96–98]. Despite models such as the collaborative care model promoted by the 2018 Lancet Commission, many LMICs remain unprepared to provide basic care for CPMDs in primary care [97, 99].

The discussion above raises the questions of *where* and *how* to begin integrating maternal mental health within the health system. Our findings suggest that there are several possible entry points. Results from existing programs, as well as the analysis of the core components of CPMD interventions at community and health systems levels, highlight five potential “places” that can be considered entry points. Several of these should be considered in an integrated manner when attempting to truly address CPMDs. The first, and arguably most important given the ability to affect the largest number of women, is the community level. This would be working through community health workers, peers, grandmothers, or new mental health cadres who women trust and who can meet women where they are [48]. The second place would be through children and family-based approaches. We found that using children as a “trojan horse” for entry, or externally focusing on children rather than women, could improve acceptability and reduce the pressure, guilt, and stigma for mothers. The third place for potential entry would be where many women already seek medical care during their perinatal period—ANC/PNC facilities. Training health care workers in CPMD prevention, care, and treatment can be incorporated into any intervention that is working within health system strengthening [55]. To integrate into any of these platforms, provider pre-service education—a fourth entry point—needs to be strengthened. Preparing the next generation of health workers to properly diagnose and treat women suffering from CPMDs early and with the care they need is an important step in the long term [84]. And the fifth and last place, a very important and often overlooked area for intervention, is within faith-based communities. Our LA found a powerful link between mental health and spirituality/faith. Collaborating with local traditional healers and faith leaders is a needed to affect true change in women’s perinatal mental health.

Although there are successful interventions and documented strategies, our findings suggest there are a number of gaps in the current literature and implementation learning that are ripe for future research. First, the LA uncovered widespread calls for more evidence on populations that are particularly vulnerable to CPMDs, such as women in humanitarian settings, adolescents, women experiencing IPV, and women experiencing perinatal loss. Often decisions are made for these women based on evidence generated in completely different contexts, limiting the benefit these women stand to gain from the services they receive. Second, there is a need for research that looks beyond postpartum depression; gaps exist in the understanding of other mental health conditions and during other stages of the perinatal period [100]. Linked to the need to better understand the context and social determinants, the LA found that what women want during the perinatal period, both in terms of outcomes and preferences among services, rarely set direction for research and program priorities. Women should be a part of defining the problems as well as the ways solutions are presented. Similarly, and even more surprisingly, the LA found very few examples of co-creation or long-term inclusivity beyond initial, one-off qualitative research. And lastly, more evidence is needed about interventions that work practically for both women’s and children’s long term health outcomes and that also clarify our understanding of health outcomes and causal pathways. The relationship between PMH and child outcomes may be part of a more complex causal pathway, and more needs to be done to understand the pathway [31]. The ability to link CPMD interventions into existing platforms that benefit women and children holds great promise for improving health outcomes, and overall quality of life, for both.

## Limitations

The scoping review may have excluded papers and grey literature because of search terms, older papers, and non-English language literature. The LA review of literature did not assess for generalizability, and findings presented may not apply to all perinatal women. Given the gap in overall research in LMICs, and the concentration of publications in certain regions, the issues many know are facing women and communities may not have been written about at all. The selection of literature for the LA focused on LMICs may have excluded relevant topics researched in higher-income settings, such as risks associated with pharmacotherapy during pregnancy [101, 102].

## Conclusion

The need to provide CPMD prevention, care, and treatment programs to women in LMICs is evident. The question of how to integrate these services in a person-centered, respectful manner that is feasible, sustainable, and scalable remains largely unanswered. Global MNCAH actors, global mental health actors, national decision-makers, and local communities have not yet come together to collectively address this critical question. Establishing and delivering maternal mental health services has not been prioritized for a number of reasons. Without written policy and designated funding at the national level, addressing needs of maternal mental health lacks a sense of urgency, and already overburdened health care workers are unable to take on more “work” to provide screening and care. The nature of mental health suffering itself also disguises the scale and severity of the burden—it is less obvious than other causes of maternal morbidity and mortality, and stigma and biases keep women and families silent—contributing to a continued focus on more visible health concerns, such as postpartum hemorrhage. But something must be done to help millions of women “suffering in silence” every day.

Despite good evidence-based interventions that have been tested in study settings, and promising low-resource interventions that have emerged from within communities, our landscape analysis identified a glaring gap in how to intervene in a low-cost, highly scalable manner that improves outcomes for both women and children across contexts. However, with core components identified, stakeholders can lead thoughtful, context-responsive adaptations to these approaches to implement high-impact programs now, while generating better evidence. Future evidence needs to: expand and include different contexts (vulnerable populations, diverse geographic coverage); look beyond postnatal depression; develop care models that are responsive to women’s expressed desires; identify integrated approaches that improve health for both women and children; be generated from the countries and communities they are going to be introduced in; and flow from evidence-based policy. As implementation research continues to improve the delivery of these interventions in real-world settings, cost-effective and feasible interventions for mental illness, with demonstrated effectiveness for CPMDs, can be implemented today [50, 103, 104].

## Supplementary Information

**Additional file 1.** Key Informant Interview Guide.

**Additional file 2.** Focus Group Discussion Guide.

## Data Availability

Anonymized qualitative data will be made available upon reasonable request to the corresponding author.

## Abbreviations

ANC: Antenatal Care
CHW: Community Health Worker
CPMD: Common Perinatal Mental Disorder
DISC: Discrimination and Stigma Scale
FGD: Focus Group Discussion
GBV: Gender-Based Violence
HCW: Health Care Worker
IPV: Intimate Partner Violence
KII: Key Informant Interview
LA: Landscape Analysis
LMIC: Low- and Middle-Income Country
MCGL: MOMENTUM Country and Global Leadership
mhGAP: Mental Health Gap Action Program
MNCAH: Maternal, Newborn, Child, and Adolescent Health
PMH: Perinatal Mental Health
PNC: Postnatal Care
RMC: Respectful Maternity Care
WHO: World Health Organization

## Footnotes

1 Countries were identified as LMICs using World Bank income classifications for 2021.

2 The countries included in this analysis were 16 countries where MCGL is currently implementing health programs plus three additional countries (with *) where there were strong examples to learn from. The countries were Bangladesh, Cameroon, Cote d’Ivoire, Ethiopia,* Ghana, India, Indonesia, Kenya, Liberia, Malawi, Nepal, Nigeria, Pakistan,* Rwanda, South Africa,* Tanzania, Togo, Uganda, and Vietnam.

3 Other interventions targeting PMH have been summarized in detail in recent systematic reviews by Rahman et al. and Gajaria et al., both in 2018.
