# Supplementary material for: The silent burden: a landscape analysis of common perinatal mental disorders in low- and middle-income countries
Source: BMC Pregnancy Childbirth. 2022 Apr 20;22:342. doi: 10.1186/s12884-022-04589-z (PMC9019797; doi:10.1186/s12884-022-04589-z)
Supplement: Supplementary file 1 — Additional file 1. Key Informant Interview Guide. [file 12884_2022_4589_MOESM1_ESM.docx]

**Additional File 1: Key Informant Interview Guide**

**Key Informant Interview Guide** – Program Implementation

**Assessment of maternal mental health (and effect on newborn/infant) in priority countries**

| Interviewee number: |  |
| --- | --- |
| Organization: |  |
| Country: |  |
| Date of interview:  Time of Interview: |  |
| Interview Conducted By: |  |
| Length of Interview: |  |
| Notes/Observations about the interview process (not the interview content) |  |

**Introduction**

Hello my name is <<XX>> and I am working with MCGL to conduct a Landscape Analysis on maternal mental health in LMICs. I am reaching out to you as you have worked, in your professional capacity, in the implementation of interventions to address maternal mental health. The aim of this interview is to better understand program and intervention implementation where you are working. You do not have to join and can refuse to answer questions at any time. If you agree, and say yes, we will ask you about maternal mental health interventions and research being conducted in LMICs. This interview will take 60 minutes. I will be recording this call’s audio – the video will be turned off. If you agree to continue, may I please have your consent to be recorded and to continue? Thank you for agreeing to discuss with me.

1. Can you please tell me the context of maternal mental health in your country (or the country you are working in)?

PROBE: Describe the maternal mental health situation in your country.

1. What are the cultural and community beliefs about mental illness?

PROBE: What are the beliefs about mental illness that are passed down from your elders?

1. What are mothers struggling with in the country/community you work in?

PROBE: What are the common challenges/difficulties for mothers in the communities you work in that go beyond pregnancy?

1. What are the risk factors for maternal mental health disorders?

PROBE: What life experiences or health concerns impact a mother's mental health?

1. How accessible are services?

PROBE: How easily can a new mother or pregnant woman see someone if she is feeling depressed/anxious?

1. Can you please describe the program/intervention that you are working on to address maternal mental health?
2. What is working well in the program/intervention?

PROBES:

- 1. What about human resources?
  2. What about cognitive behavioral therapy?
  3. What about music?
  4. What about individual counseling?
  5. What about parenting classes?
  6. What about integration?

1. How does this intervention address the risk factors?
2. What challenges has the program experienced?
   1. How has the intervention/program you are working with managed these complications?
3. How could this intervention be scaled up or replicated?
4. How does a program or intervention acknowledge and address the mental health aspects related to the complex array of issues from poverty to gender-based violence and food insecurity?
5. Is there anything else about the intervention or program adjustments made that you would like to share with me?
