# Supplementary material for: The silent burden: a landscape analysis of common perinatal mental disorders in low- and middle-income countries
Source: BMC Pregnancy Childbirth. 2022 Apr 20;22:342. doi: 10.1186/s12884-022-04589-z (PMC9019797; doi:10.1186/s12884-022-04589-z)
Supplement: Supplementary file 2 — Additional file 2. Focus Group Discussion Guide. [file 12884_2022_4589_MOESM2_ESM.docx]

**Focus Group Discussion Guide** – Program Implementation in humanitarian

settings

**Assessment of maternal mental health (and effect on newborn/infant) in priority countries**

| Interviewee numbers: |  |
| --- | --- |
| Organization: |  |
| Country: |  |
| Date of interview:  Time of Interview: |  |
| Interview Conducted By: |  |
| Length of Interview: |  |
| Notes/Observations about the interview process (not the interview content) |  |

**Introduction**

Hello my name is <<XX>> and I am working with MCGL to conduct a Landscape Analysis on maternal mental health in LMICs. I am reaching out to you all as you have worked, in your professional capacities, in the implementation of interventions to address maternal mental health within the humanitarian sector. The aim of this focus group discussion is to better understand the current maternal mental health situation as well as program and intervention experiences where you are working.

CONSENT:

*A focus group is a small discussion group of people brought together to share thoughts on a specific topic. You may choose not to answer any question that we ask. You may leave the discussion at any time. With your permission, the group session will be audio-recorded and transcribed. The information on the tapes is confidential and only the study team members will listen to them. The recordings will be destroyed after they have been transcribed. If any member of the focus group does not wish to be audio-recorded, a member of the study team will take notes instead.*

*We will ask you questions about how people who are like you [age, gender, work, etc.] think about maternal mental health in humanitarian settings. We do not want you to tell us anything personal about yourself.*

*We do our best to protect your identity and comments made during the group discussion. Comments made by others during the group discussion should not be discussed with people outside the group. We cannot guarantee that participants will keep things confidential.*

*This Focus Group Discussion will take 90 minutes. Please confirm your consent.*

Thank you for agreeing to discuss with me.

1. Can you please describe the context of maternal mental health in the humanitarian setting?

PROBE: Describe the maternal mental health situation in the countries you work in.

1. What are the main risk factors driving mental health disorders for women?

PROBE: What are the factors that contribute to women’s mental health?

1. What cultural norms exist that help women seeking and receiving care?

PROBE: What about communal society?

What about religion of traditional beliefs?

1. What cultural norms exist that prevent women from seeking and/or receiving care?

PROBE: What mental health issues are highly stigmatized?

1. What implementation strategies have worked in the humanitarian setting?

PROBE: What has made these strategies successful?

1. What implementation strategies have not worked in the humanitarian setting?

PROBE: What has caused these strategies to fail?

1. Is the humanitarian sector able to pull apart larger psychosocial support for the population from maternal health needs?
   1. If yes, how?
   2. If no, why not?
2. What needs to be done to ensure women and their mental health needs are met in this complex setting?

IF ANY EXTRA TIME:

1. What, if anything, is being done to measure the prevalence?
   - 1. To meet the needs of women?
     2. To meet the needs of newborns?
2. Are there any cultural emphases on self-reliance?
3. Are there any cultural emphases on strength?

**Focus Group Discussion Guide** – Program Implementation: working with adolescent girls in maternal health and wellbeing

**Assessment of maternal mental health (and effect on newborn/infant) in priority countries**

| Interviewee numbers: |  |
| --- | --- |
| Organization: |  |
| Country: |  |
| Date of interview:  Time of Interview: |  |
| Interview Conducted By: |  |
| Length of Interview: |  |
| Notes/Observations about the interview process (not the interview content) |  |

**Introduction**

Hello my name is <<XX>> and I am working with MCGL to conduct a Landscape Analysis on maternal mental health in LMICs. I am reaching out to you all as you have worked, in your professional capacities, in the implementation of interventions to address maternal mental health with adolescents. The aim of this focus group discussion is to better understand the current maternal mental health situation as well as program and intervention experiences where you are working.

CONSENT:

*A focus group is a small discussion group of people brought together to share thoughts on a specific topic. You may choose not to answer any question that we ask. You may leave the discussion at any time. With your permission, the group session will be audio-recorded and transcribed. The information on the tapes is confidential and only the study team members will listen to them. The recordings will be destroyed after they have been transcribed. If any member of the focus group does not wish to be audio-recorded, a member of the study team will take notes instead.*

*We will ask you questions about how people who are like you [age, gender, work, etc.] think about maternal mental health of adolescent girls in LMICs. We do not want you to tell us anything personal about yourself.*

*We do our best to protect your identity and comments made during the group discussion. Comments made by others during the group discussion should not be discussed with people outside the group. We cannot guarantee that participants will keep things confidential.*

*This Focus Group Discussion will take 90 minutes. Please confirm your consent.*

Thank you for agreeing to discuss with me.

IF ADOLESCENT Health focused

1. Can you please describe the context of maternal mental health amongst adolescent moms?

PROBE: Describe the maternal mental health situation in the countries you work in.

1. What are the main risk factors driving mental health disorders for women in emergencies/humanitarian settings?

PROBE: What are the factors that contribute to women’s mental health in emergencies/humanitarian settings??

1. How, if at all, are the adolescent girls different from women in their experiences with maternal mental health?
2. What cultural norms exist that **help** women seeking and receiving care?

PROBE: What about communal society?

What about religion or traditional beliefs?

1. What cultural norms exist that **prevent** women from seeking and/or receiving care?

PROBE: What mental health issues are highly stigmatized?

1. What implementation strategies have worked in the humanitarian setting?

PROBE: What has made these strategies successful?

1. What implementation strategies have not worked in the humanitarian setting?

PROBE: What has caused these strategies to fail?

1. Is there anything else I should know about working to improve adolescent maternal mental health?

IF THERE IS EXTRA TIME:

1. What, if anything, is being done to measure the prevalence?
   - 1. To meet the needs of adolescents?
     2. To meet the needs of their newborns?
2. How should the health system evolve to create a woman centered, inclusive space that adolescent girls would seek out?
